# A Novel Tree Shrew Model of Diabetic Retinopathy

**DOI:** 10.3389/fendo.2021.799711

**Published:** 2022-01-03

**Authors:** Oleg S. Gorbatyuk, Priyamvada M. Pitale, Irina V. Saltykova, Iuliia B. Dorofeeva, Assylbek A. Zhylkibayev, Mohammad Athar, Preston A. Fuchs, Brian C. Samuels, Marina S. Gorbatyuk

**Affiliations:** ^1^ Department of Optometry and Vision Science, School of Optometry, University of Alabama at Birmingham, Birmingham, AL, United States; ^2^ Department of Dermatology, School of Medicine, University of Alabama at Birmingham, Birmingham, AL, United States; ^3^ Department of Ophthalmology and Visual Sciences, School of Medicine, University of Alabama at Birmingham, Birmingham, AL, United States

**Keywords:** tree shrews, diabetes, diabetic retinopathy, diabetic cone, retinal ganglion cells, p-AKT/p-mTOR axis

## Abstract

Existing animal models with rod-dominant retinas have shown that hyperglycemia injures neurons, but it is not yet clearly understood how blue cone photoreceptors and retinal ganglion cells (RGCs) deteriorate in patients because of compromised insulin tolerance. In contrast, northern tree shrews (*Tupaia Belangeri*), one of the closest living relatives of primates, have a cone-dominant retina with short wave sensitivity (SWS) and long wave sensitivity (LWS) cones. Therefore, we injected animals with a single streptozotocin dose (175 mg/kg i.p.) to investigate whether sustained hyperglycemia models the features of human diabetic retinopathy (DR). We used the photopic electroretinogram (ERG) to measure the amplitudes of A and B waves and the photopic negative responses (PhNR) to evaluate cone and RGC function. Retinal flat mounts were prepared for immunohistochemical analysis to count the numbers of neurons with antibodies against cone opsins and RGC specific BRN3a proteins. The levels of the proteins TRIB3, ISR-1, and p-AKT/p-mTOR were measured with western blot. The results demonstrated that tree shrews manifested sustained hyperglycemia leading to a slight but significant loss of SWS cones (12%) and RGCs (20%) 16 weeks after streptozotocin injection. The loss of BRN3a-positive RGCs was also reflected by a 30% decline in BRN3a protein expression. These were accompanied by reduced ERG amplitudes and PhNRs. Importantly, the diabetic retinas demonstrated increased expression of TRIB3 and level of p-AKT/p-mTOR axis but reduced level of IRS-1 protein. Therefore, a new non-primate model of DR with SWS cone and RGC dysfunction lays the foundation to better understand retinal pathophysiology at the molecular level and opens an avenue for improving the research on the treatment of human eye diseases.

## Introduction

Diabetic retinopathy (DR) is on the priority list of eye conditions according to the World Health Organization. One-third of diabetes patients are affected by this disease and its prevalence is expected to triple, reaching 643 million DR patients by 2040 (1). DR is a leading cause of blindness for working-age individuals in developed countries worldwide (1, 2). Neurodegeneration and retinal vasculature dysfunction are two major pathophysiological features of DR (3). Aberrant retinal vascularization is a primary diagnostic characteristic and determinant for current DR treatments. Retinal dysfunction (4–9) caused by reduced cone sensitivity, abnormal activation of the phototransduction cascade (10, 11), selective loss of S cones (12), glial abnormalities, and thinning of the nerve fiber, the retinal ganglion cell (RGC) layer, and the inner plexiform layer in DR patients (13–15) have not received the necessary attention from researchers. Highlighting the importance of further investigation, hyperglycemia-induced retinal neurodegeneration may precede the microvascular dysfunction and contribute to DR pathogenesis (16–19). The ability to investigate crosstalk between these two pathologic features of DR depends on establishing animal models that accurately represent diabetic retinopathy pathophysiology in the human eye.

Retinal ganglion and amacrine cells are the first neurons in which hyperglycemia induces apoptosis (20). Photoreceptor cells in the diabetic retina also die through apoptosis and calpain activation (21, 22). Several animal models have been developed to study the molecular basis of DR pathogenesis. While they accurately reproduce some of the pathological changes found in patients, several critical features, such as cone photoreceptor cell degeneration (10, 12), RGC loss (23), proliferative retinopathy (24) are still missing. Non-human primate models have high structural similarity to the human eyes, but have several limitations, including the lack of cone neurodegeneration, cell loss (24), and the high cost. Similarly, rodent models do not usually manifest cone deficits. Previous studies with various models of DR lack consistent conclusions about the presence of photoreceptor loss in diabetes (25). Some studies propose that hyperglycemia induces no photoreceptor loss (4, 26), while others detected photoreceptor cell death in diabetic retinas (25, 27–29). This emphasizes the gap in the methodology and the approaches applied to study DR.

RGC death has been documented in human diabetic retinas and animal models of DR (23, 30). Severe DR significantly reduces the density of RGC and affects the expression of melanopsin in melanopsin containing RGCs (mRGCs) in the human diabetic retina, partially explaining the abnormal circadian activity and pupil responses in patients with diabetes (31, 32). Moreover, parasol and midget RGCs show irregular axon morphology, swelling, and beading with significantly reduced branching frequency, in the diabetic human retina (33). The molecular mechanism of RGC death is probably apoptosis through activation of caspase-3, FAS, and BAX found in postmortem tissues (34). Several studies with diabetic rodents have shown dramatic RGC loss as early as 6 weeks after streptozotocin (STZ) injection (35). After three months of diabetes, the hyperglycemic Ins2Akita/+ mice suffer from RGC loss in the peripheral retina with morphological changes in the dendrites of the surviving large ON-type RGCs (36). This is in line with other DR models and STZ-induced diabetes (37), although the timing of RGC death in mice varies (38–40).

The lack of DR models representative of the full clinical spectrum of humans disease, in combination with the unavailability of human diabetic retinas suitable for analysis, is probably responsible for the disregard of some DR biomarkers (e.g., metabolic retinal stress). An animal model recapitulating pathogenesis of human DR is a critical unmet need and would assist resolving these issues. Such a model would provide a platform for the development of novel therapeutics targeting photoreceptor dysfunction and retinal metabolic stress.

Diurnal tree shrews (*Tupaia Belangeri*) have cone-dominant retinas (95%). The highest cone density is located in the central retina.

Although there is no fovea in the tree shrew’s retina, the RGC density peak corresponds to the area centralis (temporal retina) and is similar to the area of central vision (41). These animals are dichromatic with 90–96% red-sensitive cones (Long Wave sensitivity or LWS) and 10% blue-sensitive cones (Short wave sensitivity or SWS), depending on their retinal location (42, 43). Together, these findings suggest that tree shrews could be a useful model of DR. Therefore, we hypothesize that tree shrews are an ideal and unique model of DR that accurately simulates critical aspects of human DR and will serve for translational research aimed at preventing vision loss or restoring vision in patients with diabetic retinopathy. This novel model could lay the foundation for a better understanding of the molecular mechanisms underlying DR pathophysiology.

## Materials and Methods

### Animals

All experiments were approved by the UAB Institutional Animal Care and Use Committee and adhered to all standards set forth in the ARVO Statement for the Use of Animals in Ophthalmic and Vision Research. Male tree shrews were purchased from the UAB tree shrew core. Tree shrews are available from the UAB core on commercial base. Animals were housed under conditions of a 12-h light-dark cycle with unlimited access to food and water, until at least 6 months of age.

Hyperglycemia was induced by a single intraperitoneally injection of 175 mg/kg of STZ (Sigma, St. Louis, MO, United States). The control group was injected with vehicle (0.09% phosphate buffer i.p.). Four weeks following the injection, the blood glucose levels (BGL) from tail blood samples were measured once a week for 16 weeks using Accu-Check Aviva Plus test strips (Roche Diabetes Care, Inc.). Detection of BGL at > 250 mg/dL for four consecutive weeks served as evidence of hyperglycemia, although we continued monitoring BGL in animals over the course of experimental procedure. All blood glucose tests were conducted in non-fasting animals at 10:00 AM. Bodyweight (BW) of control and hyperglycemic three shrews was monitored weekly. BW loss > 20% for 10 consecutive days, BGL > 350 mg/dL, and high level of ketones in urea measured by a ketone detection kit (Trivida Health, Inc.) served as exclusion criteria from the study and was considered a study endpoint.

At 16 weeks after STZ injection, diabetic and control animals were euthanized. Blood samples were collected, and biochemical analysis was conducted to determine the concentration of glycated hemoglobin (HbA1C), free fatty acids (FFA), total cholesterol (TC), high- and low-density lipoproteins (HDL and LDL, respectively), triglycerides (TG), glucagon (Gluc), and insulin (Ins) in the Vanderbilt University Medical Center Lipid Core.

### Retinal Function Test

Because tree shrews are dichromatic animals with 90-96% LWS cones and 7-10% SWS cones, depending on retinal location (42, 43), we analyzed the A- and B-waves of photopic cone-mediated ERG using the LKC UTAS-3000 Diagnostic System (Gaithersburg, MD) as described in (44). Animals were anesthetized with ketamine (100 mg/kg) and xylazine (10 mg/kg), and their pupils were dilated with topical 2.5% phenylephrine hydrochloride ophthalmic solution (Paragon BioTeck, Inc). After performing the light adaptation procedure on the background for 10 min with a light intensity of 2.5 cd*s/m^2^, 7.91 cd*s/m^2^, 25 cd*s/m^2^, and 79.1 cd*s/m^2^, we performed an ERG recording averaging 15 sweeps with the interval of 1 sec and using white light as a stimulus. ERG lens electrodes (LKC Technologies) were placed on the surfaces of both eyes by using Gonak™ 2.5% Hypromellose Ophthalmic Demulcent Solution (Akorn, Inc.). We measured the A-wave amplitudes from the baseline to the peak in the cornea-negative direction and the B-wave amplitudes from the cornea-negative peak to the major cornea-positive peak. Fifteen waveforms were recorded from each animal and the values were averaged. This protocol was also used to measure photopic negative responses (PhNR). 

### Fundus Imaging Analysis

SD-OCT imaging was performed using a Spectralis OCT2 (Heidelberg Engineering, Inc, Heidelberg, Germany) as described in (45). Animals were anesthetized with ketamine and xylazine as described above. One drop of topical 2.5% phenylephrine (Paragon BioTeck, Inc) was used to dilate the iris. SD-OCT horizontal B-scans were performed to detect changes in the retinal angiography using the sodium fluorescein dye. The Micron IV imaging system (Phoenix Technology Group, LLC) was used to register retinal blood-vessel leakage. To that end, anesthetized animals received IP injections of sodium fluorescein and the imaging was processed as described in (44).

### Histological Analysis

For the histological analysis, the eyeballs were enucleated and emersion fixed in freshly prepared 4% paraformaldehyde in phosphate-buffered saline (PBS). Hematoxylin and eosin staining of 16 μm thick retinal cryosections was performed. Digital images of diabetic and control retinas were analyzed in the central, superior, and inferior hemispheres equally distant from the optic nerve head. The thicknesses of outer nuclear layer (ONL) and inner nuclear layer (INL) on whole retinas were analyzed by an investigator blind to the experimental condition. The pancreas tissues were collected from control and diabetic animals, fixed, and processed for paraffin embedding. Tissues were then sectioned and stained with H&E prior to histopathologic analysis for morphological signs of STZ-induced damage.

To calculate cone and RGC density, we prepared retinal flat mounts. The lens and the vitreous body were carefully removed from the eyecup leaving the retinal pigment epithelium (RPE)and neuronal retinas. The neural retina was then separated from remaining RPE layer to perform immunohistochemistry. For retinal flat mounts and cryosections, the retinal tissues were rinsed with PBS, blocked with 5% normal donkey serum, and 0.3% Triton X-100 in PBS for 1 h at room temperature. The retinal flat mounts and cryosections were then incubated in the primary anti-BRN3A antibody (#ab245230, Abcam) in a dilution 1:300 to identify RGCs, anti-red/green opsin (#AB5405, Millipore), and anti-blue opsin (#AB5407, Millipore) antibodies in a dilution of 1: 200 overnight to detect LWS and SWS cones, respectively. The secondary Alexa Fluor 555 or 488 antibodies (TermoFisher Scientific) diluted in PBS (1:500) were applied for 1 h at room temperature. Retinal flat mounts were covered with coverslips after applying mounting medium with propidium iodide (#H-1300-10, Vector Laboratories, Inc). Fluorescent microscopy was performed using a BZ-X Keyence fluorescent microscope to count the numbers of red/green and blue cones in a 0.01 mm^2^ counting region of interest in the central, mid-central, and peripheral areas (zone-1, zone-2, and zone-3) of the superior regions of the eye.

### Western Blot Analysis

The tree shrew’s retinas were dissected. Lysis was carried out with RIPA buffer to prepare the protein extracts. The primary antibodies used in the study were as follows: anti-BRN3A (#ab245230, Abcam), anti-p-AKT (#p-S473, 4060), AKT (#4691), anti-p-mTOR (D9C2, 5536), mTOR (# 7C16, 2983), and anti-ISR1 (#3407) from Cell Signaling; anti-TRIB3 (B-2) (# sc-390242) from Santa-Cruz Biotechnology) and rabbit anti-Actin (# A2066), and mouse anti-β-Actin (# A2228) from Sigma-Aldrich.

### Statistics

The Student’s *t*-test was used to compare the control and the diabetic groups. All statistical analyses were performed on GraphPad Prism 9 software.

## Results

### A Single STZ Injection Resulted in Sustained Hyperglycemia in Tree Shrews

**Figure 1:**
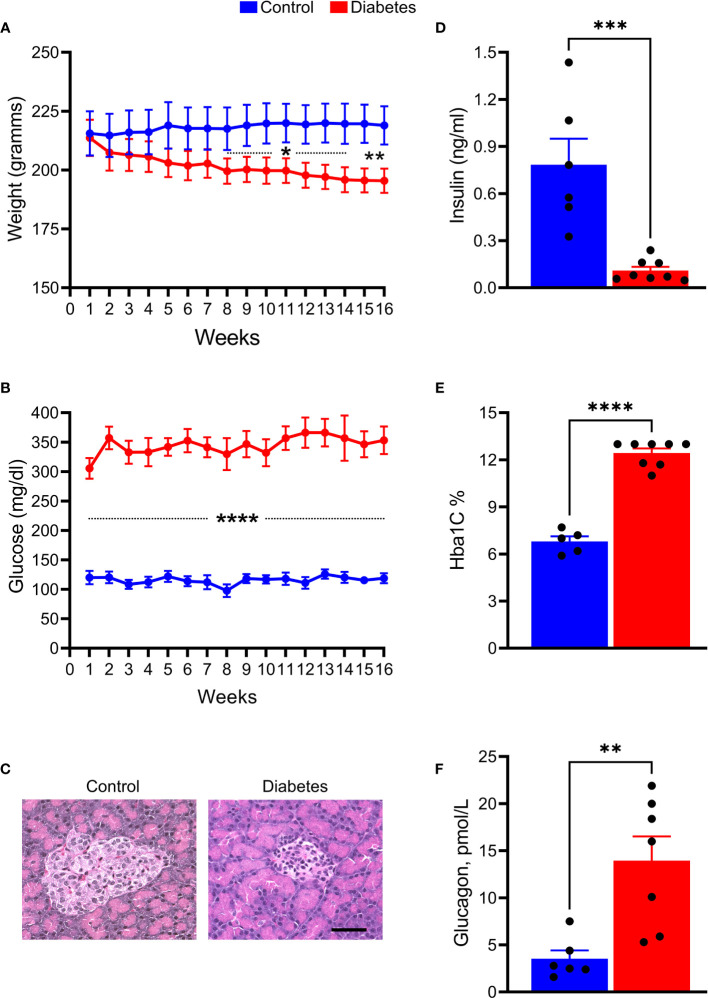
STZ injection results in the development and progression of T1D diabetes in tree shrews**. (A)** The body weight was reduced in diabetic tree shrews, starting at 8 weeks after injection. **(B)** At 1 week after the STZ injection, the blood glucose levels were significantly and consistently upregulated > 300 mg/ml in diabetic animals as compared with control tree shrews. **(C)** The islet β cells were almost lost in the pancreas of diabetic animals as a result of STZ injection leading to diminished insulin production measured in the serum of diabetic and control animals **(D, E)** The HbA1c levels measured at the end of the experimental protocol were markedly increased in diabetic tree shrews compared with control animals, suggesting sustained hyperglycemia after injection with STZ. **(F)** Both the reduction in insulin production and the increase in HbA1C were in agreement with elevated glucagon level in the serum of diabetic tree shrews (n=xx). *p < 0.05, **p < 0.01, ***p < 0.001, ****p < 0.000, n= 5-8.

Over 75% of the STZ-injected tree shrews developed type 1 diabetes (T1D). The rest of the animals did not manifest sustained hyperglycemia but most likely developed type 2 diabetes (T2D) and were excluded from the study. We monitored the animals for 16 weeks and found that these animals demonstrated a progressive decline in BW as compared to the control group, starting at 8 weeks after induction of hyperglycemia (*p* < 0.01; **Figure 1A**). BGL levels were also elevated in STZ-injected animals (**Figure 1B**). The average BGL fluctuated between 300 mg/dl and 350 mg/dl in the diabetic group during the 16 weeks of observation. Both BGL and BW values were consistent with the decline in insulin levels (8-fold, *p <*0.001) and the increase in HbA1C (2-fold, *p* < 0.0001), and Glucagon (>3-fold, p<0.01), biomarkers of T1D, measured after animals had been euthanized (**Figures 1C–F**).

**Figure 2:**
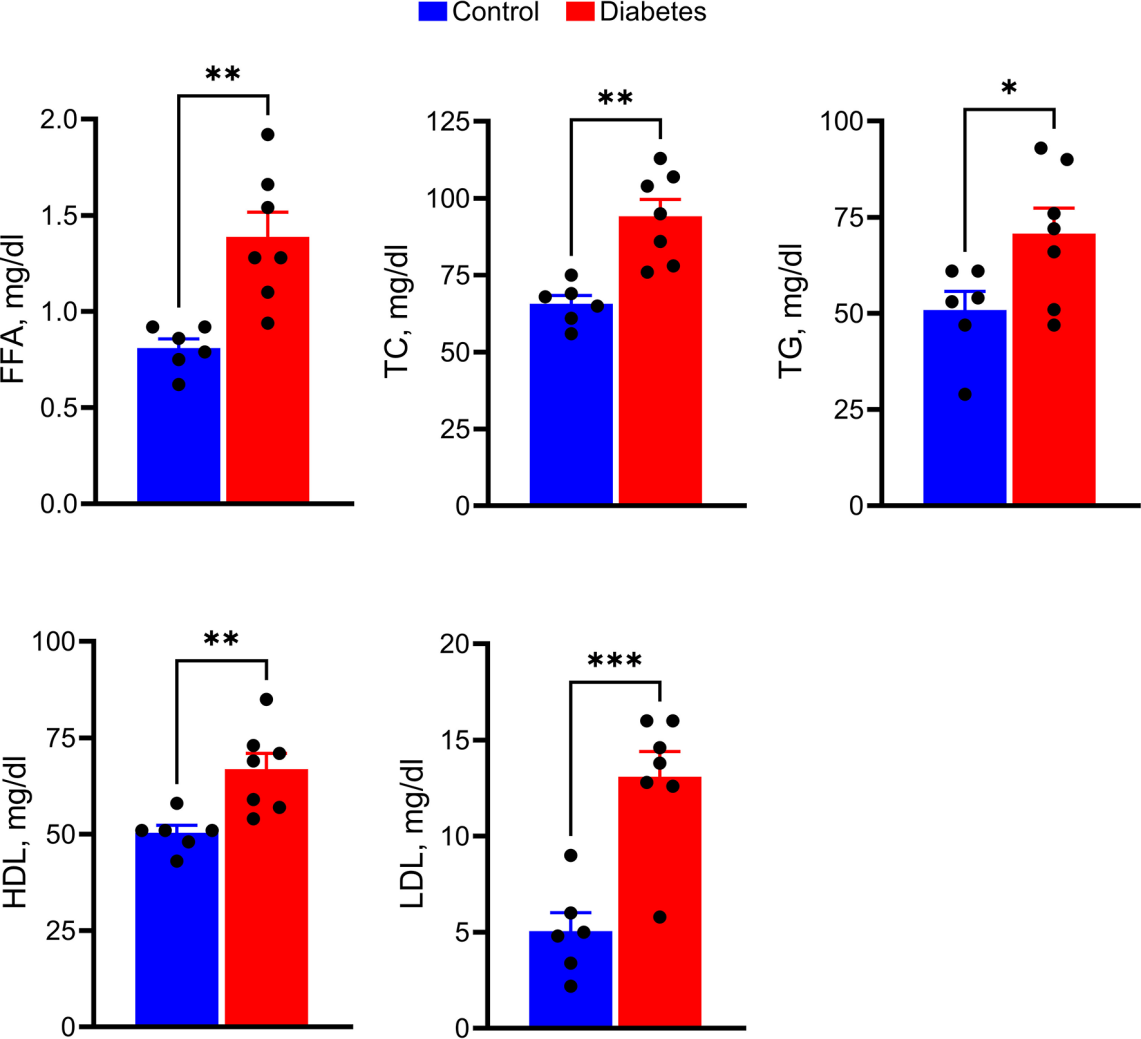
Hyperglycemic tree shrews undergo changes in the levels of serum parameters such as FFA, TC, TG, HDL, and LDL. *p < 0.05, **p < 0.01, ***p < 0.001, (n= 6-7).

Overall, tree shrew injected with a single dose of STZ developed severe and sustained hyperglycemia and were further examined for markers of lipid metabolism (**Figure 2**). Serum levels of FFA (2-fold), TC (1.4-fold), HDL (1.3-fold), LDL (> 2-fold), and TG (1.5-fold) were significantly elevated in hyperglycemic tree shrews compared to control animals.

### Hyperglycemic Tree Shrews Manifest Loss of Retinal Function

**Figure 3:**
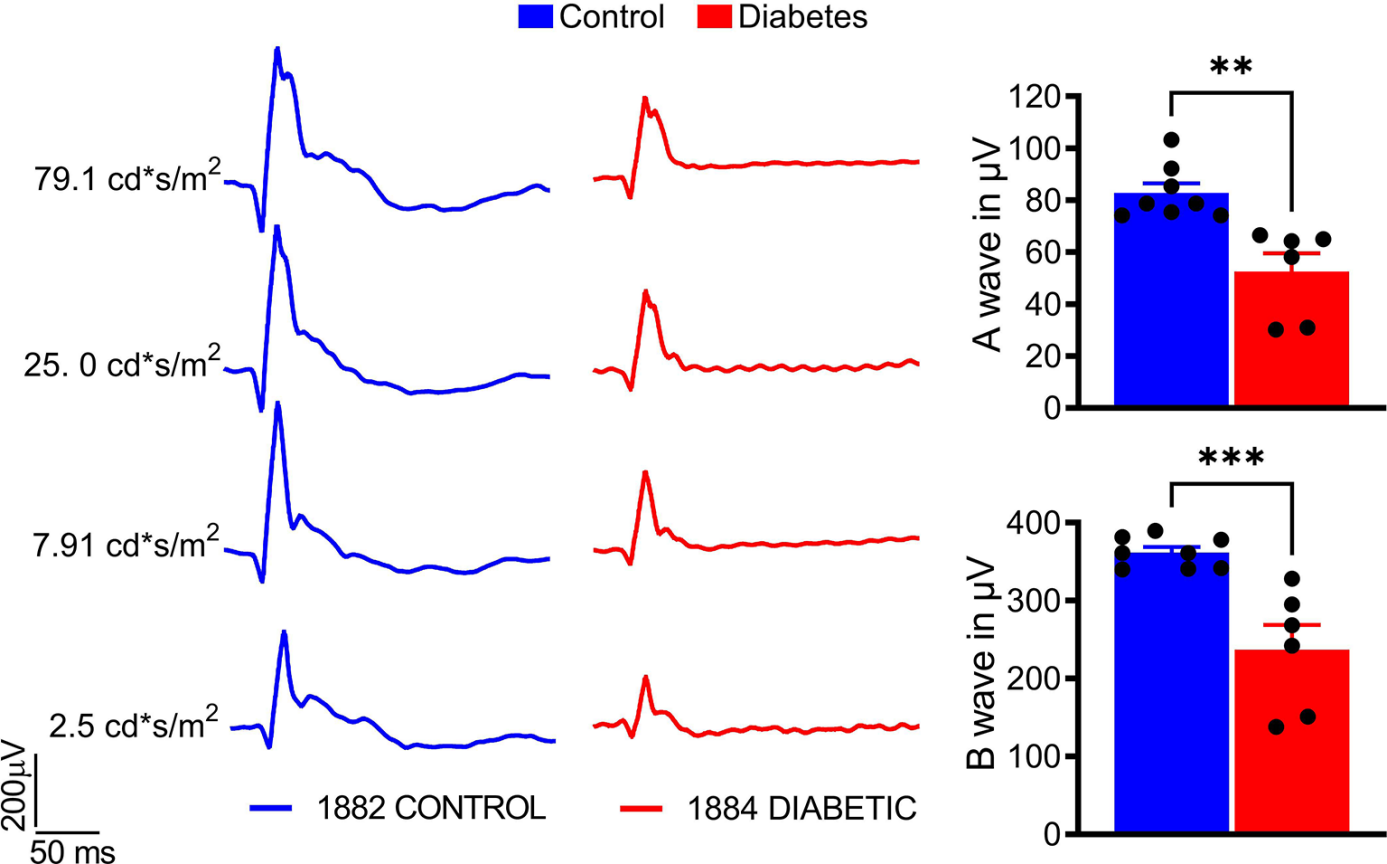
Sustained hyperglycemia results in compromised cone photoreceptor function. The photopic ERG were recorded with the LKC setup (right). A- and B-wave photopic ERG amplitudes were diminished in the diabetic tree shrew retina (left). The calculation of the photopic ERG A- and B- wave amplitudes at 25 cd*s/m^2^ is shown. **p < 0.01, ***p < 0.001, (n= 6-8).

**Figure 4:**
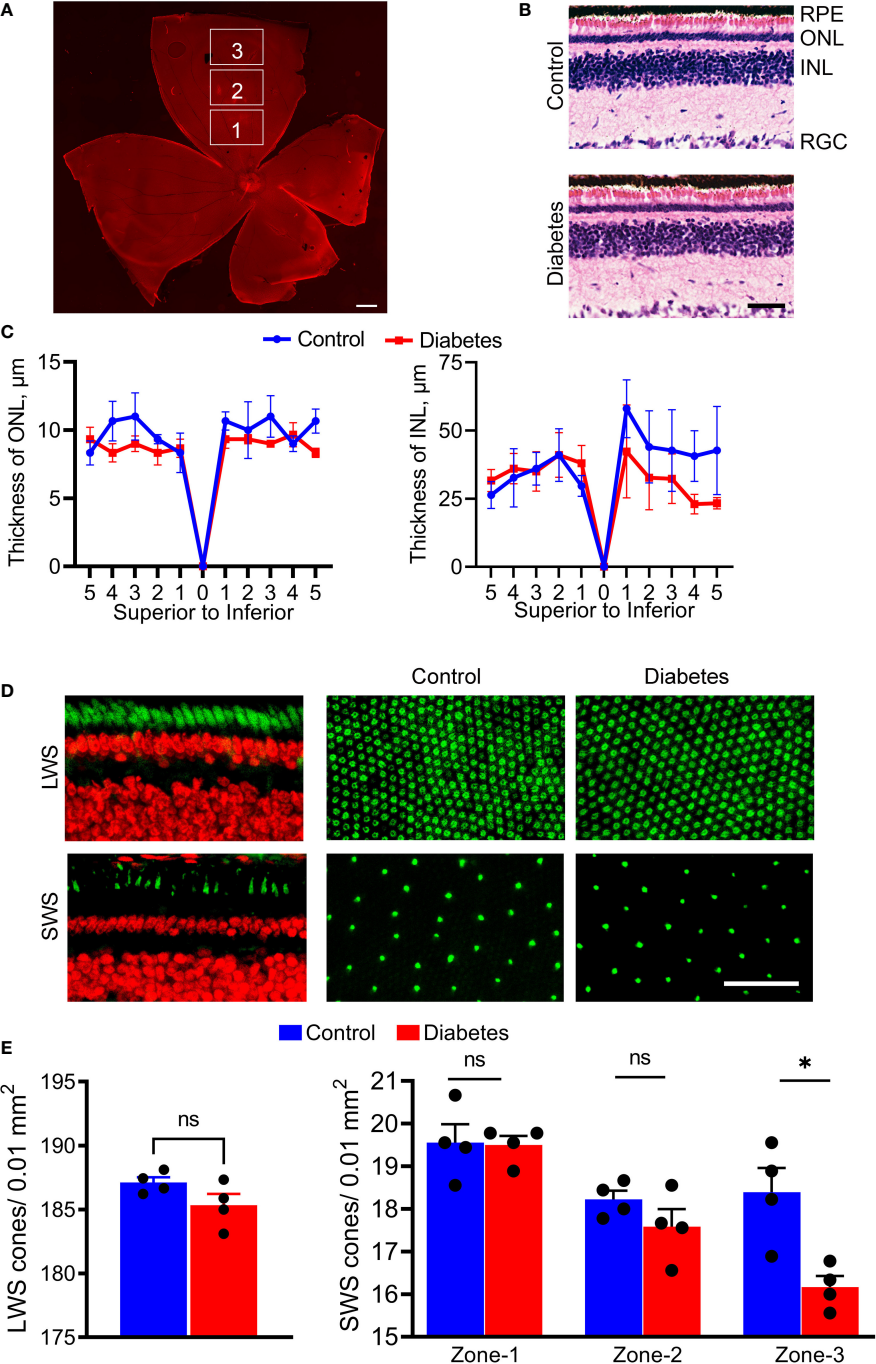
Histological changes of diabetic retinas. **(A)** The retinal flat mounts were divided by zone-1, zone-2, and zone-3 to count cone photoreceptor and retinal ganglion cells (propidium iodide in red). **(B)** Images of the H&E-stained control and diabetic retinas. **(C)** Spider plots depicting the thicknesses of the ONL and INL. The spider plots were generated by plotting the number of nuclei using 1000 µm step in the distance from the ONH for both hemispheres. **(D)** Fluorescent images showing sections of the retina and retinal flat mounts from control and diabetic tree shrews processed with anti-green/red cone opsin (Green, upper panel) and anti-blue cone opsin (Green, bottom panel) primary antibodies, as well as with propidium iodide (Red). **(E)** The number of LWS cones did not differ between the diabetic and control groups (left graph); however, the number of SWS cones showed small but significant differences between diabetic and control groups in zone 3 (right graph) . *p < 0.05, (n= 4, each). Scale bars: **(A)**- 1000 µm, **(B, D)** – 50 µm. ns, non-significant.

A significant decline by 50% in both A- and B-wave amplitudes was observed in diabetic animals compared with the control group (*p* < 0.01 and *p* < 0.001, respectively; **Figure 3**), indicating retinal deterioration. Therefore, we next analyzed the number of cones and the thicknesses of the Outer and Inner Nuclear Layers (ONL and INL, respectively) across the retina (**Figure 4**).

The stitching of fluorescent images allowed us to divide superior retinas into central (zone 1), mid-peripheral (zone 2), and peripheral (zone 3) sectors (**Figure 4A**) and count the number of cones. While the decrease in ONL and INL thicknesses did not reach statistical significance in diabetic retinas at this time point (**Figures 4B, C**), in zone 3, there was a significant (12%) decrease in the number of SWS cones between control and diabetic retinas (*p* < 0.0004, **Figures 4D, E**). Central and mid-peripheral retinas had no significant differences between the two groups. Moreover, the decrease in the number of SWS cones was not accompanied by loss of LWS cones across the entire retina (**Figures 4D, E**), suggesting a selective peripheral SWS cone sensitivity to hyperglycemia.

**Figure 5:**
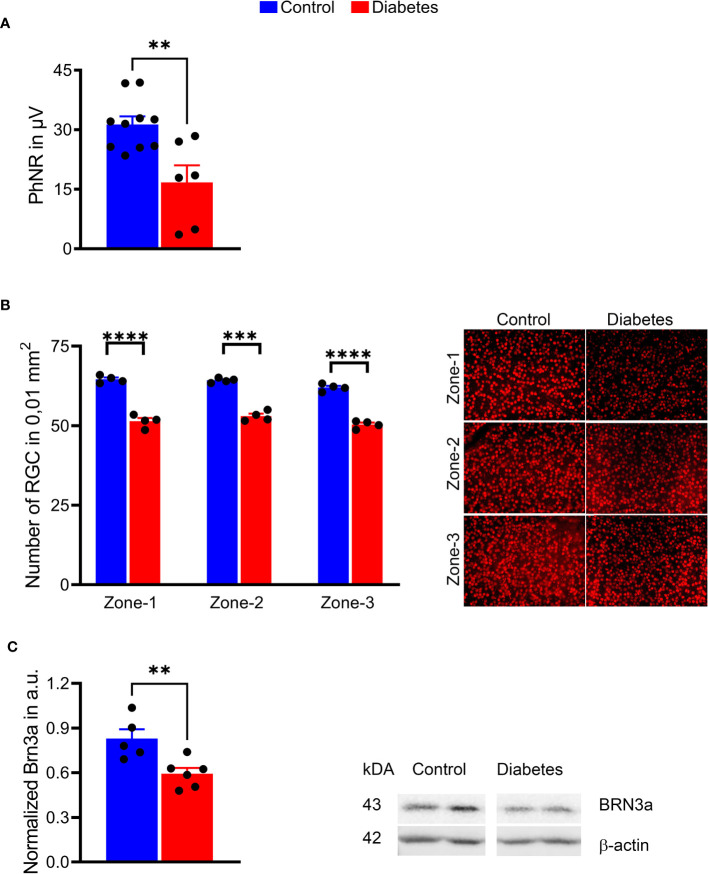
Sustained hyperglycemia resulted in compromised RGC function. **(A)** The PhNR amplitudes were reduced by 50% in the diabetic retina (right panel). **(B)** Images of the retinal flat mount processed with anti-BRN3A antibody (left panel). The number of BRN3A-positive RGCs in the diabetic retina was markedly reduced in zone 1, zone 2, and (zone-3 areas compared to the control group (right panel). See also Figure 4 for depicted area. **(C)** In agreement with the immunohistochemistry analysis, the expression of BRN3A protein was also diminished as detected by western blot analysis (representative membrane images on the left; calculated data on the right). **p < 0.01, ***p < 0.001, ****p < 0.0001, (n= 5-10).

Because of the RGC loss in patients (33) and RGC death in diabetic animals described in our and other published studies (23, 37), we analyzed the PhNRs and calculated the number of RGCs in diabetic retinal flat mounts (**Figure 5**). A 2-fold decline in PhNR was observed in the diabetic group as compared to control animals (*p* < 0.001), suggesting a compromised RGC function after 16 weeks of sustained hyperglycemia (**Figure 5A**, see also **Figure 3A**). A marked reduction in BRN3A-positive RGCs was found in all three sectors of retinal flat mounts. This reduction was in the range of 17–20% (*p* < 0.0001 for all sectors) (**Figure 5B**). Interestingly, the observed RGC loss was in agreement with BRN3A protein levels in diabetic retinas (**Figure 5C**), where BRN3A protein levels were reduced by 29%, as evaluated by western blot analysis. These results suggest that sustained hyperglycemia compromised retinal function and induced RGC and peripheral SWS cone loss in diabetic tree shrews.

### Diabetic Tree Shrews Do Not Manifest Vascular Leakage After 16 Weeks of Hyperglycemia

After the detection of the neuronal deficits in diabetic retinas, we tested for signs of vascular abnormalities in the hyperglycemic tree shrews by fluorescein angiography. Neither vascular leakage nor retinal vessel abnormalities were observed after 16 weeks of hyperglycemia (**Figure S1**). This finding suggests that the retinal cell function loss was not accompanied by compromised vascular health in diabetic tree shrews.

### Sustained Hyperglycemia Correlates With the Upregulation of the p-AKT/p-mTOR Pathway in the Diabetic Tree Shrew’s Retina

**Figure 6:**
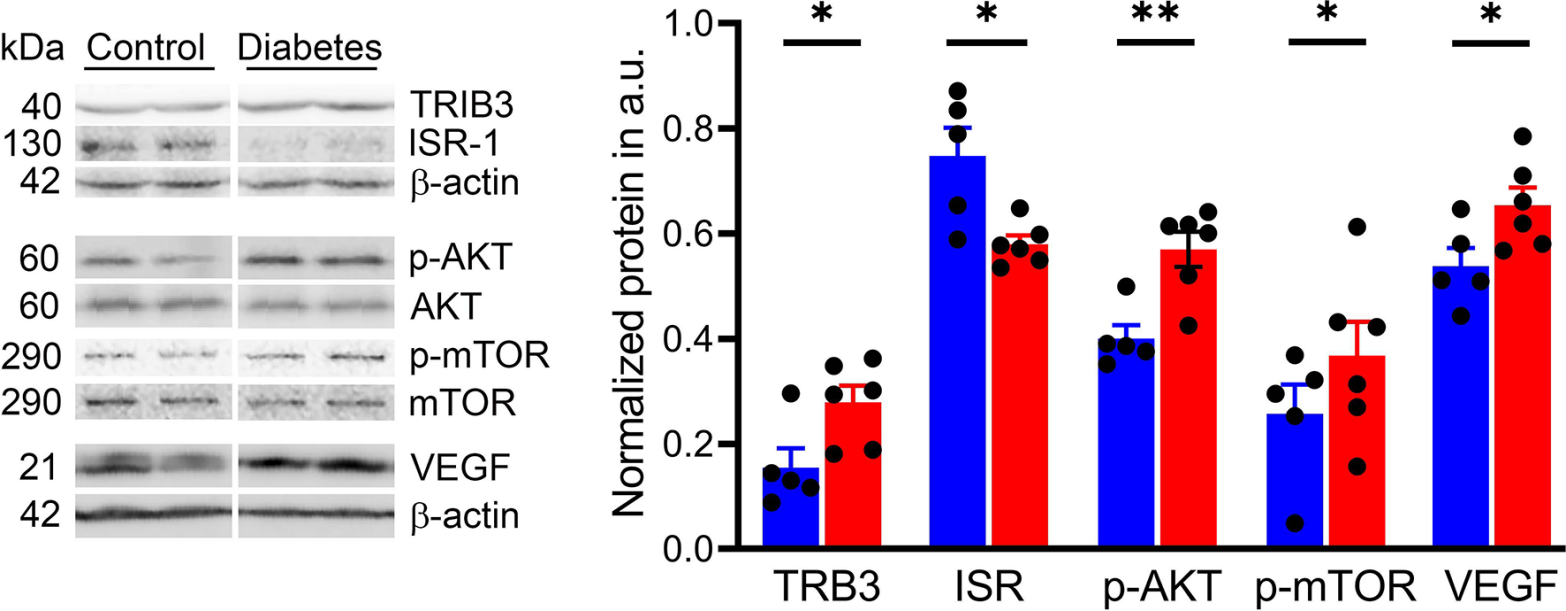
Sustained hyperglycemia results in the alteration of cellular signaling in diabetic retinas. Representative images of the western blot membranes are shown on the left. The measured normalized values of the proteins in question are shown on the right. Reduced IRS expression was detected in diabetic retinas along with an increase in the p-AKT/p-mTOR pathway. In addition, we observed an increase in TRIB3 and VEGF expression, supporting our previous findings in human and mouse diabetic retinas. *p < 0.05, **p < 0.01, (n= 5-6).

We next investigated the activity of the ISR/AKT/mTOR pathway in the retina of the cone-dominant diabetic tree shrew. The insulin signal receptor (ISR) was significantly downregulated in diabetic retinas compared with control animals (*p* < 0.05; **Figure 6**). This decrease was associated with the 1.4-fold increase in both p-AKT and p-mTOR (*p* < 0.01 and *p* < 0.05, respectively). Because of our recent findings suggesting the regulation of p-AKT/p-mTOR by the pseudokinase TRIB3 in mice with inherited retinal degeneration (46) and the TRIB3 upregulation in human and mouse diabetic retinas (37), we tested the TRIB3 levels as well. Similar to diabetic patients and mice, diabetic tree shrews’ retinas showed an upregulation of TRIB3 (**Figure 6**). This upregulation was associated with a 20% increase in the level of VEGFP.

## Discussion

The results of the present study indicate that tree shrews develop sustained hyperglycemia and manifest type 1 diabetic biomarkers and circulating markers of lipid metabolism dysfunction as well as loss of retinal function and peripheral SWS cone and global RGC death at 16 weeks after STZ injection.

STZ is a widely used pharmacological compound applied to induce experimental diabetes mellitus. However, the experimental doses of STZ and the severity and onset of diabetes vary across species. Here, we provided the evidence that a single injection of 175 mg/kg STZ in tree shrews leads to sustained hyperglycemia and, eventually, culminates in T1D and diabetic retinopathy.

Cone photoreceptor dysfunction is a clinical manifestation of DR (10, 12), and a substantial decrease in parafoveal cone density has been reported in patients with non-proliferative DR (9, 47). Despite the reports on cone photoreceptor degeneration in patients with DR, some controversy still exists over the relationship between cone density decline and HbA1c levels (9, 47). Here, we report that increased HbA1C and Glucagon and reduced insulin levels correlate with retinal neuronal dystrophy and SWS cone photoreceptor cell death.

A recent study on basal physiological parameters suggested that tree shrews could be an ideal animal model for metabolic diseases (48). Since spontaneous diabetes in tree shrews has many similarities to human diabetes (49), tree shrews could be an attractive model for DR. However, to date, diabetes research in tree shrews has been limited to the study of the liver or kidneys and has not yet focused on the retina (50–52). Here, we show that hyperglycemic tree shrews manifest changes in circulating FFA, TC, HDL, LDL, and TG (**Figure 2**).

Although FFA is an important link between obesity, insulin resistance, and type 2 diabetes, little is known about their levels in T1D. A recent study conducted in patients with T1D identified an increase in the ratio of saturated to unsaturated FFA compared to nondiabetic controls (53). Despite this increase, total FFA in the serum of T1D patients was lower compared to the control group (**Figure 2**). Our study, in contrast, indicates an increase in total FFA in diabetic tree shrews compared with the control group. This could be explained, in part, by the particular metabolic processes of tree shrews or the need for controlled clinical studies of diabetes in patients. For example, normal tree shrews have a high basal HDL/LDL ratio compared to humans (54). Therefore, it is not surprising that both HDL and LDL respond to hyperglycemia. Interestingly, the above-mentioned study also provided evidence for a positive correlation between FFA and cholesterol and HDL. Evidence from our study showed that diabetic tree shrews also manifested a positive correlation between FFA and TC, FFA, HDL, and BGL further supporting this notion.

The retinal changes in STZ-injected animals are likely due to hyperglycemia and not STZ toxicity. Indeed, direct toxicity of STZ on lymphocytes, particularly on CD8+ cells, Treg, and B cells have been previously shown *in vitro* (55). Although STZ, a toxic glucose analog, targets pancreatic β-cells *via* GLUT2 transporter uptake, a great deal of the literature suggests that expression of GLUT2 is more robust in the pancreas than in the retina (56). Next, considering the very short half-life of STZ (approximately 15 min) (57), it is unlikely that its direct effect could be sustained for a few months after the administration. Therefore, most likely that the observed functional, morphological, and molecular changes in injected animals are due to STZ-induced hyperglycemia. These changes include minor but significant loss of peripheral SWS cones, similar to the S cone damage found in patients with T1D (12, 58). Thus, we confirmed that the tree shrew retina contains SWS cones at a percentage of approximately 10% of the total number of cones (**Figures 4**). The decline in the cone-derived photopic ERG amplitudes in the diabetic group is in agreement with this finding. However, given that the reduction in LWS cones, a major cone type in the tree shrew’s retina, is not observed, most likely, the ERG abnormalities arise from dysfunction of the cones in diabetic retinas. This hyperglycemia-induced dysfunction of photoreceptors has been previously reported (59). Therefore, together with the observed decline in RGC function and cell death detected in patients and diabetic animals, this feature indicates the neuronal deficit in hyperglycemic retinas. Future studies investigating prolonged hyperglycemia (>16 weeks) will help determine whether SWS cone loss remains isolated to the periphery or migrates slowly toward the central retina surrounding the optic nerve head. Additionally, these studies will help determine if LWS cones remain resistant to damage caused by prolonged hyperglycemia.

Notably, the lack of compromised retinal vascular health in diabetic tree shrews despite a 20% increase in VEGF at this timepoint indicates that the neuronal retinal pathogenesis most likely occurs before major vascular damage, making it a limitation of the diabetic tree shrew model for advanced stages of DR in humans. Indeed, the retinal neurons could be more vulnerable to hyperglycemia as compared to retinal endothelial cells. In turn, signal transmission from deteriorating retinal neurons together with elevated glucose, diminished insulin, and variations in the circulating lipids, lipoproteins, and proteins collectively may damage the vascular tissue at later stages of diabetes. Therefore, the selected experimental period (16 weeks) could be the reason why we did not observe vascular changes with non-invasive imaging analysis yet.

The deficit in circulating insulin is in agreement with reduced ISR-1 levels in diabetic retinas. This finding is in agreement with previous studies in diabetic rats after 12 weeks of hyperglycemia (60). However, in the rat model, the authors observed a decline in the AKT activity, while our study showed an upregulation of the p-AKT/p-mTOR pathway after 16 weeks of hyperglycemia. Interestingly, TRIB3, a metabolic stress indicator, is elevated in response to hyperglycemia as documented in another study by our group on diabetic humans and mice (37). However, the TRIB3-mediated control of p-AKT in the retina of mice with inherited retinal degeneration did not coincide with the present results on the diabetic retina, implying that the cellular signaling may be differentially activated depending on the type of stress.

## Conclusions

We propose a new tree shrew model of DR with the retinal phenotypes of cone photoreceptor degeneration and RGC dysfunction to investigate the hyperglycemia-induced retinal degeneration at early stages of DR. This model also lays the groundwork for better understanding molecular pathophysiology of DR and opens an avenue for improving the treatment of human eye disease. Moreover, this model can be an ideal bridge between the non-human primate and rodent models of diabetes. Finally, by simulating critical pathophysiological aspects of human DR, this model could also serve to evaluate the effect of systemic pathogenesis of human diabetes, including the affected pancreas, liver, and kidneys on the development and progression of DR.

## Data Availability Statement

The raw data supporting the conclusions of this article will be made available by the authors, without undue reservation.

## Ethics Statement

The animal study was reviewed and approved by UAB Institutional Animal Care and Use Committee

## Author Contributions

OG and MG designed the study. OG, IS, PP, ID, and AZ performed the experiments. BS and PF analyzed diabetic animals by SD-OCT. BS, MA, OG, and MG discussed the experimental results. OG and MG wrote the manuscript. All authors contributed to the article and approved the submitted version.

## Funding

This work was supported by the National Eye Institute, grants R21EY031103, R01027763 and the UAB Vision Science P30 Core (P30EY003039).

## Conflict of Interest

The authors declare that the research was conducted in the absence of any commercial or financial relationships that could be construed as a potential conflict of interest.

## Publisher’s Note

All claims expressed in this article are solely those of the authors and do not necessarily represent those of their affiliated organizations, or those of the publisher, the editors and the reviewers. Any product that may be evaluated in this article, or claim that may be made by its manufacturer, is not guaranteed or endorsed by the publisher.

## Supplementary Material

Supplementary Figure 1 Images of Micron IV fluorescein (right) and Spectralis OCT2 (left) angiograms taken from control **(A, B)** and diabetic **(C, D)** tree shrews.

## References

[1] Wong TY Sabanayagam C. Strategies to Tackle the Global Burden of Diabetic Retinopathy: From Epidemiology to Artificial Intelligence. Ophthalmologica (2020) 243:9–20. doi: 10.1159/000502387 31408872

[2] Resnikoff S Pascolini D Etya’ale D Kocur I Pararajasegaram R Pokharel GP. Global Data on Visual Impairment in the Year 2002. Bull World Health Organ (2004) 82:844–51. PMC2623053 15640920

[3] Rossino MG Dal Monte M Casini G. Relationships Between Neurodegeneration and Vascular Damage in Diabetic Retinopathy. Front Neurosci (2019) 13:1172. doi: 10.3389/fnins.2019.01172 31787868 PMC6856056

[4] Barber AJ Lieth E Khin SA Antonetti DA Buchanan AG Gardner TW. Neural Apoptosis in the Retina During Experimental and Human Diabetes. Early Onset Effect Insulin J Clin Invest (1998) 102:783–91. doi: 10.1172/JCI2425 PMC508941 9710447

[5] Murakami T Yoshimura N. Structural Changes in Individual Retinal Layers in Diabetic Macular Edema. J Diabetes Res (2013) 2013:920713. doi: 10.1155/2013/920713 24073417 PMC3773460

[6] Juen S Kieselbach GF. Electrophysiological Changes in Juvenile Diabetics Without Retinopathy. Arch Ophthalmol (1990) 108:372–5. doi: 10.1001/archopht.1990.01070050070033 2310337

[7] Di Leo MA Caputo S Falsini B Porciatti V Greco AV Ghirlanda G. Presence and Further Development of Retinal Dysfunction After 3-Year Follow Up in IDDM Patients Without Angiographically Documented Vasculopathy. Diabetologia (1994) 37:911–6. doi: 10.1007/BF00400947 7806021

[8] Tyrberg M Lindblad U Melander A Lovestam-Adrian M Ponjavic V Andreasson S. Electrophysiological Studies in Newly Onset Type 2 Diabetes Without Visible Vascular Retinopathy. Doc Ophthalmol (2011) 123:193–8. doi: 10.1007/s10633-011-9298-6 22057379

[9] Soliman MK Sadiq MA Agarwal A Sarwar S Hassan M Hanout M. High-Resolution Imaging of Parafoveal Cones in Different Stages of Diabetic Retinopathy Using Adaptive Optics Fundus Camera. PloS One (2016) 11:e0152788. doi: 10.1371/journal.pone.0152788 27057752 PMC4825992

[10] McAnany JJ Park JC. Cone Photoreceptor Dysfunction in Early-Stage Diabetic Retinopathy: Association Between the Activation Phase of Cone Phototransduction and the Flicker Electroretinogram. Invest Ophthalmol Vis Sci (2019) 60:64–72. doi: 10.1167/iovs.18-25946 30640972 PMC6333111

[11] Holopigian K Greenstein VC Seiple W Hood DC Carr RE. Evidence for Photoreceptor Changes in Patients With Diabetic Retinopathy. Invest Ophthalmol Vis Sci (1997) 38:2355–65. 9344359

[12] Cho NC Poulsen GL Ver Hoeve JN Nork TM. Selective Loss of S-Cones in Diabetic Retinopathy. Arch Ophthalmol (2000) 118:1393–400. doi: 10.1001/archopht.118.10.1393 11030822

[13] Verbraak FD. Neuroretinal Degeneration in Relation to Vasculopathy in Diabetes. Diabetes (2014) 63:3590–2. doi: 10.2337/db14-0888 25342732

[14] Verma A Rani PK Raman R Pal SS Laxmi G Gupta M. Is Neuronal Dysfunction an Early Sign of Diabetic Retinopathy? Microperimetry and Spectral Domain Optical Coherence Tomography (SD-OCT) Study in Individuals With Diabetes, But No Diabetic Retinopathy. Eye (Lond) (2009) 23:1824–30. doi: 10.1038/eye.2009.184 19648899

[15] Vujosevic S Midena E. Retinal Layers Changes in Human Preclinical and Early Clinical Diabetic Retinopathy Support Early Retinal Neuronal and Muller Cells Alterations. J Diabetes Res (2013) 2013:905058. doi: 10.1155/2013/905058 23841106 PMC3694491

[16] Stem MS Gardner TW. Neurodegeneration in the Pathogenesis of Diabetic Retinopathy: Molecular Mechanisms and Therapeutic Implications. Curr Med Chem (2013) 20:3241–50. doi: 10.2174/09298673113209990027 PMC4071765 23745549

[17] Tonade D Liu H Palczewski K Kern TS. Photoreceptor Cells Produce Inflammatory Products That Contribute to Retinal Vascular Permeability in a Mouse Model of Diabetes. Diabetologia (2017) 60:2111–20. doi: 10.1007/s00125-017-4381-5 PMC5660634 28755268

[18] Tonade D Liu H Kern TS. Photoreceptor Cells Produce Inflammatory Mediators That Contribute to Endothelial Cell Death in Diabetes. Invest Ophthalmol Vis Sci (2016) 57:4264–71. doi: 10.1167/iovs.16-19859 PMC5015981 27548900

[19] Liu H Tang J Du Y Saadane A Tonade D Samuels I. Photoreceptor Cells Influence Retinal Vascular Degeneration in Mouse Models of Retinal Degeneration and Diabetes. Invest Ophthalmol Vis Sci (2016) 57:4272–81. doi: 10.1167/iovs.16-19415 PMC5015983 27548901

[20] Simo R Stitt AW Gardner TW. Neurodegeneration in Diabetic Retinopathy: Does It Really Matter? Diabetologia (2018) 61:1902–12. doi: 10.1007/s00125-018-4692-1 PMC6096638 30030554

[21] Yu F Ko ML Ko GY. Decreased MicroRNA-150 Exacerbates Neuronal Apoptosis in the Diabetic Retina. Biomedicines (2021) 9(9):1135. doi: 10.3390/biomedicines9091135 PMC8469350 34572320

[22] Saadane A Du Y Thoreson WB Miyagi M Lessieur EM Kiser J. Photoreceptor Cell Calcium Dysregulation and Calpain Activation Promote Pathogenic Photoreceptor Oxidative Stress and Inflammation in Prodromal Diabetic Retinopathy. Am J Pathol (2021) 191:1805–21. doi: 10.1016/j.ajpath.2021.06.006 PMC8579242 34214506

[23] Kern TS Barber AJ. Retinal Ganglion Cells in Diabetes. J Physiol (2008) 586:4401–8. doi: 10.1113/jphysiol.2008.156695 PMC2614025 18565995

[24] Robinson R Barathi VA Chaurasia SS Wong TY Kern TS. Update on Animal Models of Diabetic Retinopathy: From Molecular Approaches to Mice and Higher Mammals. Dis Model Mech (2012) 5:444–56. doi: 10.1242/dmm.009597 PMC3380708 22730475

[25] Kern TS Berkowitz BA. Photoreceptors in Diabetic Retinopathy. J Diabetes Investig (2015) 6:371–80. doi: 10.1111/jdi.12312 PMC4511295 26221514

[26] Enzsoly A Szabo A Kantor O David C Szalay P Szabo K. Pathologic Alterations of the Outer Retina in Streptozotocin-Induced Diabetes. Invest Ophthalmol Vis Sci (2014) 55:3686–99. doi: 10.1167/iovs.13-13562 24845643

[27] Rakoczy EP Ali Rahman IS Binz N Li CR Vagaja NN de Pinho M. Characterization of a Mouse Model of Hyperglycemia and Retinal Neovascularization. Am J Pathol (2010) 177:2659–70. doi: 10.2353/ajpath.2010.090883 PMC2966820 20829433

[28] Batenburg WW Verma A Wang Y Zhu P van den Heuvel M van Veghel R. Combined Renin Inhibition/(Pro)Renin Receptor Blockade in Diabetic Retinopathy–A Study in Transgenic (Mren2)27 Rats. PloS One (2014) 9:e100954. doi: 10.1371/journal.pone.0100954 24968134 PMC4072720

[29] Lu ZY Bhutto IA Amemiya T. Retinal Changes in Otsuka Long-Evans Tokushima Fatty Rats (Spontaneously Diabetic Rat)–Possibility of a New Experimental Model for Diabetic Retinopathy. Jpn J Ophthalmol (2003) 47:28–35. doi: 10.1016/S0021-5155(02)00631-7 12586175

[30] Amato R Lazzara F Chou TH Romano GL Cammalleri M Dal Monte M. Diabetes Exacerbates the Intraocular Pressure-Independent Retinal Ganglion Cells Degeneration in the DBA/2J Model of Glaucoma. Invest Ophthalmol Vis Sci (2021) 62:9. doi: 10.1167/iovs.62.9.9 PMC8267218 34232257

[31] Obara EA Hannibal J Heegaard S Fahrenkrug J. Loss of Melanopsin-Expressing Retinal Ganglion Cells in Patients With Diabetic Retinopathy. Invest Ophthalmol Vis Sci (2017) 58:2187–92. doi: 10.1167/iovs.16-21168 28399269

[32] Jain M Devan S Jaisankar D Swaminathan G Pardhan S Raman R. Pupillary Abnormalities With Varying Severity of Diabetic Retinopathy. Sci Rep (2018) 8:5636. doi: 10.1038/s41598-018-24015-9 29618794 PMC5884827

[33] Meyer-Rusenberg B Pavlidis M Stupp T Thanos S. Pathological Changes in Human Retinal Ganglion Cells Associated With Diabetic and Hypertensive Retinopathy. Graefes Arch Clin Exp Ophthalmol (2007) 245:1009–18. doi: 10.1007/s00417-006-0489-x 17186260

[34] Abu-El-Asrar AM Dralands L Missotten L Al-Jadaan IA Geboes K. Expression of Apoptosis Markers in the Retinas of Human Subjects With Diabetes. Invest Ophthalmol Vis Sci (2004) 45:2760–6. doi: 10.1167/iovs.03-1392 15277502

[35] Xiao A Zhou Q Shao Y Zhong HF. Effect of Intravitreal Injection of Ranibizumab on Retinal Ganglion Cells and Microvessels in the Early Stage of Diabetic Retinopathy in Rats With Streptozotocin-Induced Diabetes. Exp Ther Med (2017) 13:3360–8. doi: 10.3892/etm.2017.4431 PMC5450683 28587414

[36] Gastinger MJ Kunselman AR Conboy EE Bronson SK Barber AJ. Dendrite Remodeling and Other Abnormalities in the Retinal Ganglion Cells of Ins2 Akita Diabetic Mice. Invest Ophthalmol Vis Sci (2008) 49:2635–42. doi: 10.1167/iovs.07-0683 18515593

[37] Pitale PM Saltykova IV Adu-Agyeiwaah Y Calzi SL Satoh T Akira S. Tribbles Homolog 3 Mediates the Development and Progression of Diabetic Retinopathy. Diabetes (2021) 70:1738–53. doi: 10.2337/db20-1268 PMC8385618 33975909

[38] Kim SJ Yoo WS Choi M Chung I Yoo JM Choi WS. Increased O-GlcNAcylation of NF-kappaB Enhances Retinal Ganglion Cell Death in Streptozotocin-Induced Diabetic Retinopathy. Curr Eye Res (2016) 41:249–57. doi: 10.3109/02713683.2015.1006372 25835259

[39] Martin PM Roon P Van Ells TK Ganapathy V Smith SB. Death of Retinal Neurons in Streptozotocin-Induced Diabetic Mice. Invest Ophthalmol Vis Sci (2004) 45:3330–6. doi: 10.1167/iovs.04-0247 15326158

[40] Yang Y Mao D Chen X Zhao L Tian Q Liu C. Decrease in Retinal Neuronal Cells in Streptozotocin-Induced Diabetic Mice. Mol Vis (2012) 18:1411–20. PMC3369894 22690119

[41] Petry HM Bickford ME. The Second Visual System of The Tree Shrew. J Comp Neurol (2018) 527:679–93. doi: 10.1002/cne.24413 PMC6093812 29446088

[42] Muller B Peichl L. Topography of Cones and Rods in the Tree Shrew Retina. J Comp Neurol (1989) 282:581–94. doi: 10.1002/cne.902820409 2723153

[43] Petry HM Harosi FI. Visual Pigments of the Tree Shrew (Tupaia Belangeri) and Greater Galago (Galago Crassicaudatus): A Microspectrophotometric Investigation. Vision Res (1990) 30:839–51. doi: 10.1016/0042-6989(90)90053-N 2385925

[44] Bhootada Y Kotla P Zolotukhin S Gorbatyuk O Bebok Z Athar M. Limited ATF4 Expression in Degenerating Retinas With Ongoing ER Stress Promotes Photoreceptor Survival in a Mouse Model of Autosomal Dominant Retinitis Pigmentosa. PloS One (2016) 11:e0154779. doi: 10.1371/journal.pone.0154779 27144303 PMC4856272

[45] Samuels BC Siegwart JT Zhan W Hethcox L Chimento M Whitley R. A Novel Tree Shrew (Tupaia Belangeri) Model of Glaucoma. Invest Ophthalmol Vis Sci (2018) 59:3136–43. doi: 10.1167/iovs.18-24261 PMC6018453 30025140

[46] Saltykova IV Elahi A Pitale PM Gorbatyuk OS Athar M Gorbatyuk MS. Tribbles Homolog 3-Mediated Targeting the AKT/mTOR Axis in Mice With Retinal Degeneration. Cell Death Dis (2021) 12:664. doi: 10.1038/s41419-021-03944-w 34215725 PMC8253859

[47] Lombardo M Parravano M Lombardo G Varano M Boccassini B Stirpe M. Adaptive Optics Imaging of Parafoveal Cones in Type 1 Diabetes. Retina (2014) 34:546–57. doi: 10.1097/IAE.0b013e3182a10850 23928676

[48] Wang J Xu XL Ding ZY Mao RR Zhou QX Lu LB. Basal Physiological Parameters in Domesticated Tree Shrews (Tupaia Belangeri Chinensis). Dongwuxue Yanjiu (2013) 34:E69–74. doi: 10.3724/SP.J.1141.2013.E02E69 23572369

[49] Rabb GB Getty RE Williamson WM Lombard LS. Spontaneous Diabetes Mellitus in Tree Shrews, Urogale Everetti. Diabetes (1966) 15:327–30. doi: 10.2337/diab.15.5.327 5327707

[50] Wu X Xu H Zhang Z Chang Q Liao S Zhang L. Transcriptome Profiles Using Next-Generation Sequencing Reveal Liver Changes in the Early Stage of Diabetes in Tree Shrew (Tupaia Belangeri Chinensis). J Diabetes Res (2016) 2016:6238526. doi: 10.1155/2016/6238526 27069931 PMC4812456

[51] Zhang L Wu X Liao S Li Y Zhang Z Chang Q. Tree Shrew (Tupaia Belangeri Chinensis), A Novel Non-Obese Animal Model of Non-Alcoholic Fatty Liver Disease. Biol Open (2016) 5:1545–52. doi: 10.1242/bio.020875 PMC5087676 27659689

[52] Pan XH Yang XY Yao X Sun XM Zhu L Wang JX. Bone-Marrow Mesenchymal Stem Cell Transplantation to Treat Diabetic Nephropathy in Tree Shrews. Cell Biochem Funct (2014) 32:453–63. doi: 10.1002/cbf.3037 24867093

[53] Sobczak AIS Pitt SJ Smith TK Ajjan RA Stewart AJ. Lipidomic Profiling of Plasma Free Fatty Acids in Type-1 Diabetes Highlights Specific Changes in Lipid Metabolism. Biochim Biophys Acta Mol Cell Biol Lipids (2021) 1866:158823. doi: 10.1016/j.bbalip.2020.158823 33010452 PMC7695620

[54] Liu HR Wu G Zhou B Chen BS. Low Cholesteryl Ester Transfer Protein and Phospholipid Transfer Protein Activities Are the Factors Making Tree Shrew and Beijing Duck Resistant to Atherosclerosis. Lipids Health Dis (2010) 9:114. doi: 10.1186/1476-511X-9-114 20937151 PMC2964723

[55] Muller YD Golshayan D Ehirchiou D Wyss JC Giovannoni L Meier R. Immunosuppressive Effects of Streptozotocin-Induced Diabetes Result in Absolute Lymphopenia and a Relative Increase of T Regulatory Cells. Diabetes (2011) 60:2331–40. doi: 10.2337/db11-0159 PMC3161310 21752956

[56] Kermorvant-Duchemin E Pinel AC Lavalette S Lenne D Raoul W Calippe B. Neonatal Hyperglycemia Inhibits Angiogenesis and Induces Inflammation and Neuronal Degeneration in the Retina. PloS One (2013) 8:e79545. doi: 10.1371/journal.pone.0079545 24278148 PMC3836846

[57] Lee JH Yang SH Oh JM Lee MG. Pharmacokinetics of Drugs in Rats With Diabetes Mellitus Induced by Alloxan or Streptozocin: Comparison With Those in Patients With Type I Diabetes Mellitus. J Pharm Pharmacol (2010) 62:1–23. doi: 10.1211/jpp.62.01.0001 20722995

[58] Nomura R Terasaki H Hirose H Miyake Y. Blue-On-Yellow Perimetry to Evaluate S Cone Sensitivity in Diabetics. Ophthalmic Res (2000) 32:69–72. doi: 10.1159/000055592 10754437

[59] Tanvir Z Nelson RF DeCicco-Skinner K Connaughton VP. One Month of Hyperglycemia Alters Spectral Responses of the Zebrafish Photopic Electroretinogram. Dis Model Mech (2018) 11. doi: 10.1242/dmm.035220 PMC6215424 30158110

[60] Reiter CE Wu X Sandirasegarane L Nakamura M Gilbert KA Singh RS. Diabetes Reduces Basal Retinal Insulin Receptor Signaling: Reversal With Systemic and Local Insulin. Diabetes (2006) 55:1148–56. doi: 10.2337/diabetes.55.04.06.db05-0744 16567541

