# Supplementary figures and images for: A Novel Tree Shrew Model of Diabetic Retinopathy
Source: Front Endocrinol (Lausanne). 2022 Jan 3;12:799711. doi: 10.3389/fendo.2021.799711 (PMC8762304; doi:10.3389/fendo.2021.799711)

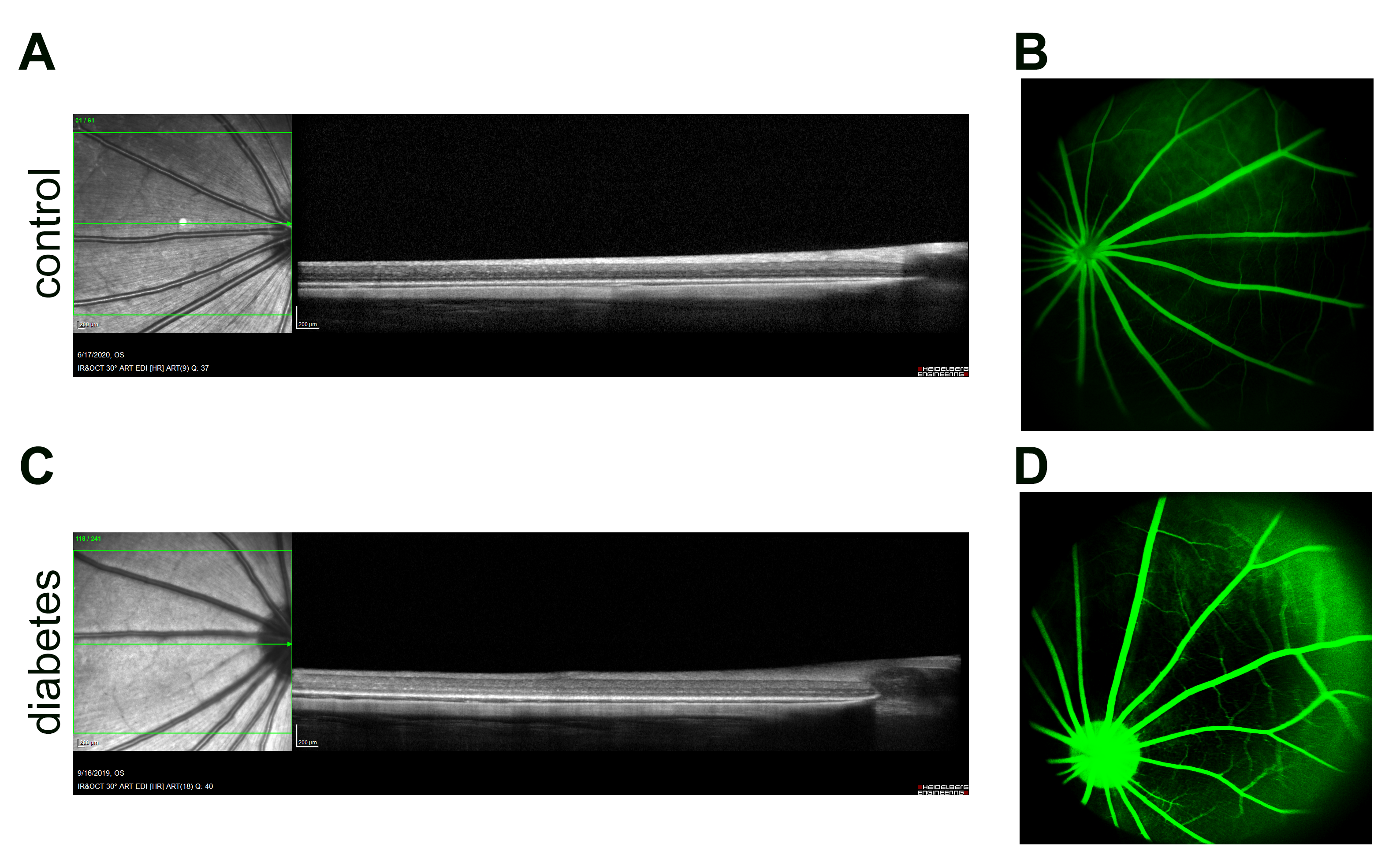

Supplement: Supplementary Figure 1 — Images of Micron IV fluorescein (right) and Spectralis OCT2 (left) angiograms taken from control (A, B) and diabetic (C, D) tree shrews. [file Image_1.tif]
